# Target trial emulation: Do antimicrobials or gastrointestinal nutraceuticals prescribed at first presentation for acute diarrhoea cause a better clinical outcome in dogs under primary veterinary care in the UK?

**DOI:** 10.1371/journal.pone.0291057

**Published:** 2023-10-04

**Authors:** Camilla Pegram, Karla Diaz-Ordaz, Dave C. Brodbelt, Yu-Mei Chang, Sarah Tayler, Fergus Allerton, Lauren Prisk, David B. Church, Dan G. O’Neill

**Affiliations:** 1 Pathobiology and Population Sciences, The Royal Veterinary College, Hatfield, Herts, United Kingdom; 2 Department of Statistical Science, University College London, London, United Kingdom; 3 Research Support Office, The Royal Veterinary College, Hatfield, Herts, United Kingdom; 4 Clinical Sciences and Services, The Royal Veterinary College, Hatfield, Herts, United Kingdom; 5 Willows Veterinary Centre & Referral Centre, Solihull, United Kingdom; Texas A&M University College Station, UNITED STATES

## Abstract

Target trial emulation applies design principles from randomised controlled trials to the analysis of observational data for causal inference and is increasingly used within human epidemiology. Veterinary electronic clinical records represent a potentially valuable source of information to estimate real-world causal effects for companion animal species. This study employed the target trial framework to evaluate the usefulness on veterinary observational data. Acute diarrhoea in dogs was used as a clinical exemplar. Inclusion required dogs aged ≥ 3 months and < 10 years, presenting for veterinary primary care with acute diarrhoea during 2019. Treatment strategies were: 1. antimicrobial prescription compared to no antimicrobial prescription and 2. gastrointestinal nutraceutical prescription compared to no gastrointestinal nutraceutical prescription. The primary outcome was clinical resolution (defined as no revisit with ongoing diarrhoea within 30 days from the date of first presentation). Informed from a directed acyclic graph, data on the following covariates were collected: age, breed, bodyweight, insurance status, comorbidities, vomiting, reduced appetite, haematochezia, pyrexia, duration, additional treatment prescription and veterinary group. Inverse probability of treatment weighting was used to balance covariates between the treatment groups for each of the two target trials. The risk difference (RD) of 0.4% (95% CI -4.5% to 5.3%) was non-significant for clinical resolution in dogs treated with antimicrobials compared with dogs not treated with antimicrobials. The risk difference (RD) of 0.3% (95% CI -4.5% to 5.0%) was non-significant for clinical resolution in dogs treated with gastrointestinal nutraceuticals compared with dogs not treated with gastrointestinal nutraceuticals. This study successfully applied the target trial framework to veterinary observational data. The findings show that antimicrobial or gastrointestinal prescription at first presentation of acute diarrhoea in dogs causes no difference in clinical resolution. The findings support the recommendation for veterinary professionals to limit antimicrobial use for acute diarrhoea in dogs.

## Introduction

Causal research methods are increasingly used within human epidemiological studies [1]. In basic terms, causal inference may refer to the process of drawing a conclusion that a specific treatment or exposure (i.e., intervention) was the “cause” of the effect (or outcome) that was observed [2]. Observational data (e.g. veterinary clinical records) are increasingly recognised as a valuable source of information to estimate real-world causal effects, especially in the absence of available randomised experiments (or in complement), and may provide a more realistic representation of a clinically relevant environment [3].

Causal inference from large observational databases (Big Data) can be viewed as an attempt to emulate the randomised experiment—the target experiment or target trial—that one would have liked to run to answer a specific question of interest [4]. Hernán and Robins (2016) have described a framework for research into comparative effectiveness using Big Data that aims to make the target trial explicit. In broad terms, the framework explicitly defines the “target trial” as the trial one would conduct if it were feasible and then describes how to emulate this target trial using observational data [4].

The VetCompass database contains large volumes of anonymised veterinary clinical data shared by UK veterinary practices that have the potential for analysis to answer a wide range of clinical causal questions. Such questions should be both clinically important and based on common conditions relevant to veterinarians in primary-care practice [5]. Diarrhoea is a commonly diagnosed condition in dogs, with one study reporting diarrhoea as the sixth most common disorder diagnosed in dogs under primary veterinary care in the UK in a one year period [5], with most cases considered mild and self-limiting [6]. Acute diarrhoea is commonly defined as lasting 7–14 days s [6–9], however, the majority (78.0%) of acute diarrhoea cases in dogs are reported to last for two days or shorter [10].

Dietary indiscretion is incriminated in the majority of acute diarrhoea cases in dogs, with affected animals generally responding well to supportive treatment [11]. Such treatment may include nutritional management, gastrointestinal nutraceutical and fluid therapy, with antimicrobials only recommended for dogs showing signs, or at high risk, of sepsis [6, 11–15]. Indeed, there is limited evidence for the clinical effectiveness of antimicrobials compared to placebo or gastrointestinal nutraceuticals for treatment of acute, uncomplicated diarrhoea [16, 17]. A large observational study including 2,429 dogs identified no association between prescription of antimicrobials and clinical resolution [18]. Although a large sample size doesn’t offset the limitations of observational studies, which generally report associations rather than causality [19], the absence of an improved response following antimicrobial therapy is in line with results from 5 prospective (randomised controlled) treatment trials [20–24].

Despite the lack of evidence for clinical effectiveness, antimicrobial prescription has been reported in over 50% of dogs with acute uncomplicated diarrhoea within 10 days of first veterinary presentation [18]. In these antimicrobial-treated cases, metronidazole was the most commonly prescribed systemic antimicrobial (47.0% of antimicrobial prescribing cases), followed by clavulanic acid potentiated amoxicillin (22.7%) [18]. Antimicrobial resistance (AMR) is considered one of the most serious and imminent health-related problems worldwide [15, 25], with indiscriminate antimicrobial use reportedly exacerbating the issue [26, 27]. As well as intra-species concerns with AMR, companion animals may act as a source of antimicrobial resistant enteric bacteria or resistance genes for their owners, given common closely shared owner-animal environments [26, 28–30]. Metronidazole specifically can also have long-term detrimental implications for the gut microbiome and metabolome, particularly in relation to bile acid dysfunction [17, 22, 31, 32]. Therefore, given the current available evidence, high prescription rates of antimicrobials for acute diarrhoea is concerning [17].

Studies evaluating the effectiveness of antimicrobials in the treatment of acute diarrhoea in dogs have also variably included gastrointestinal nutraceuticals (including prebiotics, probiotics, synbiotics, adsorbents, and motility modifiers) as a treatment group [18, 20, 21] or evaluated gastrointestinal nutraceuticals versus placebo only [33–36]. Some studies reported possible or minor acceleration in clinical resolution of canine acute diarrhoea with gastrointestinal nutraceutical treatment [33–36]. Singleton et al. (2019) found that prescription of gastrointestinal nutraceuticals, in combination with dietary modification, were positively associated with resolution of signs (OR 2.8, 95% CI 1.3–6.1), whereas Shmalberg et al. (2019) identified no significant difference in clinical resolution of diarrhoea in dogs treated with a gastrointestinal nutraceutical compared with placebo [18, 20]. This variability was highlighted in a systematic review, which pointed toward a very limited and possibly clinically unimportant effect for gastrointestinal nutraceuticals in the prevention or treatment of acute gastrointestinal disease [37]. Singleton et al. (2019) additionally evaluated the effect of anti-inflammatories, gastrointestinal agents, endoparasiticides and endectocides and dietary modification advice on diarrhoea resolution, with no significant associations identified for any of these treatments [18].

Using anonymised veterinary clinical data from the VetCompass Programme [38], the current study used the target trial framework on veterinary primary care observational data. Specifically, this study aimed to separately compare the effects on clinical resolution (defined as no revisit with ongoing diarrhoea within 30 days of first presentation) and time to treatment escalation for two common clinical management approaches: 1. antimicrobial prescription compared to no antimicrobial prescription and (separately) 2. gastrointestinal nutraceutical prescription compared to no gastrointestinal nutraceutical prescription for acute diarrhoea in dogs. This study aims to address the research gap for the clinical effectiveness of antimicrobials for acute diarrhoea in dogs [16, 17]. Given the high rates of antimicrobial prescription within treatment plans for acute diarrhoea in dogs [18] and growing concerns over AMR [15, 25], this study will provide causal evidence to guide prescribing practices by veterinary professionals. Additionally, the evidence for the clinical effectiveness of gastrointestinal nutraceuticals in the treatment of acute, uncomplicated diarrhoea in dogs is conflicting [37]. Therefore, this study can provide further evidence based on primary-care data as to whether gastrointestinal nutraceuticals have a clinical benefit or not. We hypothesized that there is no difference in “clinical resolution” or time to treatment escalation in dogs prescribed antimicrobials versus no antimicrobials or in dogs prescribed gastrointestinal nutraceuticals versus no gastrointestinal nutraceutical. Since the study utilises observational data, target trial emulation and causal inference analyses were adopted to attenuate as many sources of bias as possible.

## Materials and methods

### Data source and power calculation

The Veterinary Companion Animal Surveillance System (VetCompass), established in the UK in 2010, focuses on generating an improved evidence base from analysis of veterinary clinical data to support improved companion animal welfare [39]. VetCompass shares de-identified electronic patient record (EPR) data from over 1800 primary-care, referral, charity and emergency-care veterinary practices distributed throughout the UK for epidemiological research [38]. EPR data are extracted from practice management systems using integrated clinical queries [40] and uploaded to a secure VetCompass structured query language (SQL) database [41]. The acute diarrhoea study population included all available dogs under primary veterinary care at clinics participating in the VetCompass Programme during 2019. Dogs under veterinary care were defined as those with at least one EPR (free-text clinical note, treatment or bodyweight) recorded during 2019. Available data fields included a unique animal identifier along with species, breed, date of birth, sex, neuter status, insurance status and bodyweight, and also clinical information from free-form text clinical notes and treatment with relevant dates.

Since the study used the target trial emulation method [4], power calculations were based on an equivalence trial designed to demonstrate that a) antimicrobial therapy is equivalent to but not superior or inferior to no antibiotic therapy and b) gastrointestinal nutraceutical therapy is equivalent to but not superior or inferior to no gastrointestinal nutraceutical therapy for the treatment of acute diarrhoea in dogs [42]. This necessitated a sample size of 79 dogs in each arm. The calculation assumed 95% of cases in each treatment group resolved [18], 80% power, a 1:1 ratio of dogs prescribed antimicrobials or gastrointestinal nutraceuticals to dogs not prescribed antimicrobials or gastrointestinal nutraceuticals, a drop-out rate of 25% and a margin on the risk difference scale of 0.1 [18, 43], using risk calc Sample Size Calculator (https://riskcalc.org/samplesize/). Ethics approval was obtained from the RVC Social Science Ethical Review Board (reference number SR2018-1652).

### Case definition, case finding and covariates

Only incident cases of diarrhoea were included in the study, with these cases defined as dogs that were diagnosed with acute diarrhoea between January 1, 2019 and December 31, 2019 without any recorded history of diarrhoea in the preceding 30 days within the available EPRs. In line with VetCompass methods used in several previous studies [5, 44–46], candidate diarrhoea cases were identified applying search terms relevant to the diagnosis and management of acute diarrhoea in the clinical notes (including “acute d+”, diarr*, D+, Protexin, Prokolin, loose stools and gastroenteritis). The search findings were merged, and a random subset of these candidate cases had their clinical notes examined manually in detail to identify dogs that met the case definition. The case definition comprised dogs aged ≥ 3 months and < 10 years first diagnosed with diarrhoea by the attending veterinarian during 2019. Ages to enrol based on consultation with internal medicine specialists and previous clinical trial reports [20, 21, 23]. Exclusion criteria comprised: diarrhoea due to acute haemorrhagic diarrhoea syndrome, parvovirus, endocrinopathy (including an acute flare-up of a chronic condition), liver disease, kidney disease, immune-mediated disease, pancreatitis, parasitic cause or non-steroidal anti-inflammatory drug (NSAID) use; antimicrobial or gastrointestinal nutraceutical prescription within 30 days prior to date of acute diarrhoea diagnosis; diarrhoea for greater than 7 days prior to first diagnosis; clinical dehydration (defined by admission for intravenous fluids); hospitalisation at first presentation for acute diarrhoea and death or euthanasia at first presentation for acute diarrhoea.

**Fig 1:**
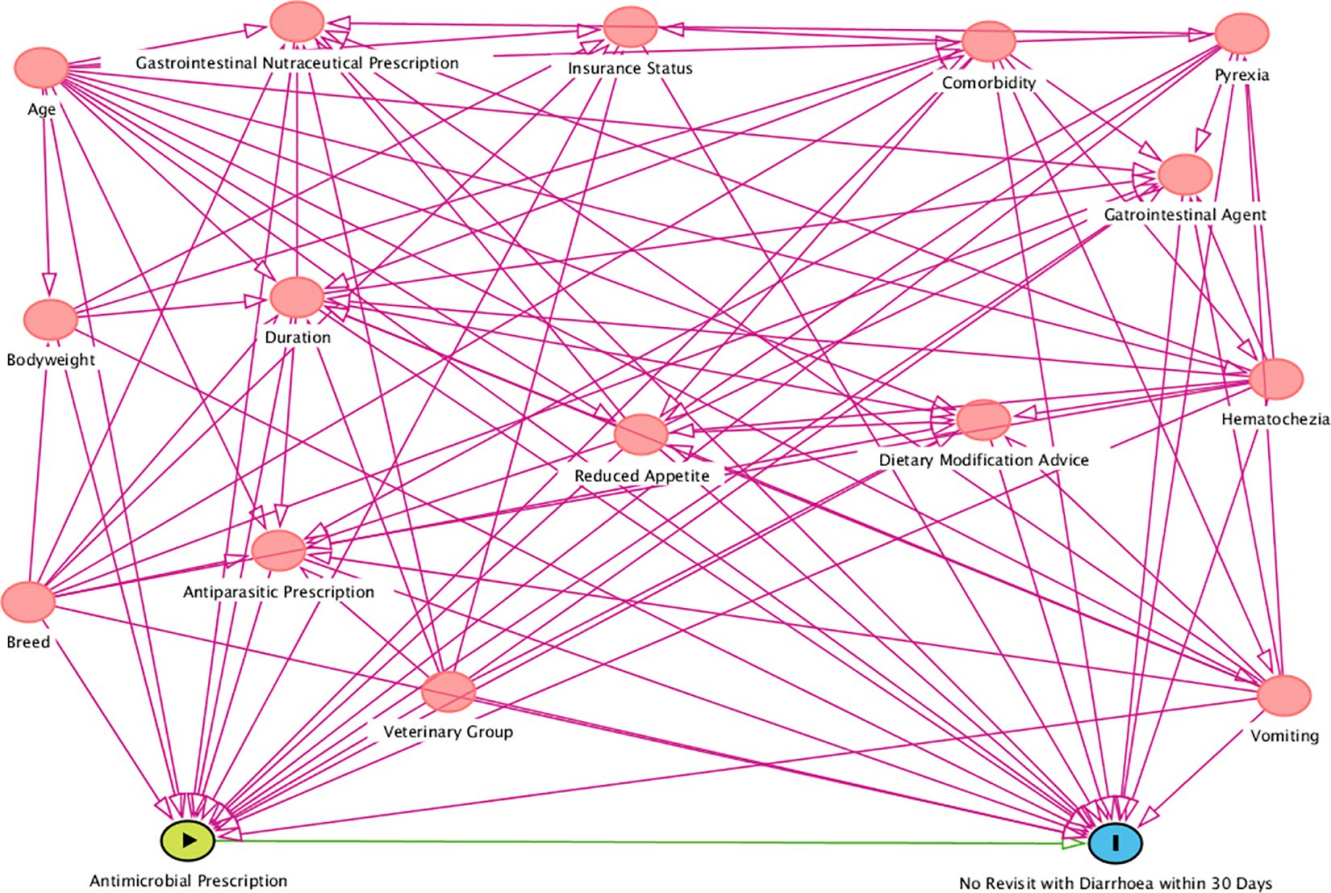
Directed acyclic graph (DAG) based on existing evidence and expert knowledge to estimate our assumptions regarding the overall effect of prescription of antimicrobials for acute diarrhoea in dogs on clinical resolution.

Based on existing evidence and expert knowledge, a directed acyclic graph (DAG) was constructed using DAGitty version 3.0, Fig 1, that encapsulated prior beliefs by the research team about the causal relationships relevant to the two questions of interest. The same covariate sets were believed to apply for both “Antimicrobial Prescription” and “Gastrointestinal Nutraceutical Prescription” as alternative exposures and “Clinical Resolution” and “Time to Treatment Escalation” as outcomes, therefore these variables replaced each other in the modelling. The DAG was used to identify which variables should be controlled for in the modelling [47], and therefore data on the following variables were collected: age, breed, bodyweight, insurance status, comorbidities, vomiting, reduced appetite, haematochezia, pyrexia, duration, additional treatment prescription and veterinary group (Fig 1). Clinical resolution (defined as no revisit with ongoing diarrhoea within 30 days of first presentation) was evaluated as the primary outcome, with time to treatment escalation a secondary outcome (with the same covariate set applied).

For dogs meeting the diarrhoea case definition, demographic data were extracted automatically from the VetCompass database, with further data relating to clinical management extracted manually from the EPR (Table 1).

**Table 1:** Definition and categorisation of demographic and clinical data extracted from the electronic patient records of dogs with acute diarrhoea attending primary-care veterinary practices in the VetCompass™ programme in the UK (n = 894).

Data extracted	Definition	Categorisation
Age	Age at first presentation of acute diarrhoea (years).	Included as continuous variable.
Breed	Breed information entered by the participating practices was cleaned and mapped to a VetCompass breed list derived and extended from the VeNom Coding breed list [48].	Antimicrobial trial: all specific breeds with at least 10 dogs prescribed antimicrobials or 15 dogs not prescribed antimicrobials, to allow sufficient study power, with remaining dogs grouped as either “Purebred–Other” or “Crossbred”. Gastrointestinal nutraceutical trial: all specific breeds with at least 15 dogs prescribed gastrointestinal nutraceuticals or 10 dogs not prescribed gastrointestinal nutraceuticals, with remaining dogs grouped as either “Purebred–Other” or “Crossbred”.
Bodyweight	The bodyweight (kg) value closest (and prior) to first presentation for acute diarrhoea.	“< 10kg”, “10 to < 20kg”, “20 to < 30kg”, “≥ 30kg” or “not recorded”.
Insurance status	Status at the final available electronic patient record.	“Insured” or “Non-insured”.
Comorbidity	Presence of at least one comorbidity recorded in the electronic patient record at or within one month prior to first presentation for acute diarrhoea.	“Yes” or “No”.
Vomiting	Concurrent vomiting reported in the electronic patient record at first presentation with acute diarrhoea.	“Yes” or “No”.
Reduced appetite	Reduced appetite reported in the electronic patient record at first presentation for acute diarrhoea.	“Yes” or “No”.
Haematochezia	Haematochezia reported in the electronic patient record at first presentation for acute diarrhoea.	“Yes” or “No”.
Pyrexia	A temperature of > 39.2°C or pyrexia (or synonym) reported in the electronic patient record at first presentation for acute diarrhoea.	“Yes” or “No”.
Duration	Duration of diarrhoea prior to first presentation as documented in the electronic patient record. Dogs with no evidence of chronic diarrhoea, but without a specific time frame reported in the EPR, were categorised as “not recorded”.	“< 24 hours”, “24–48 hours”, “48–96 hours”, “5–7 days”, or “Not recorded”.
Veterinary group	Individual practices in the study population are all part of a larger “practice group”. The practice groups were assigned a number and the group attended by each individual dog in the study recorded.	Categorised as 1–5.
Treatment prescription	Prescription of the following treatments at first presentation of acute diarrhoea: antimicrobials (oral or injectable), gastrointestinal nutraceutical (defined, based on a previous publication, as products not listed as either authorised veterinary or human medicinal products which contained a range of probiotics, prebiotics, kaolin etc., and were marketed for the purpose of aiding diarrhoea resolution [18]), dietary modification advice (defined by recommendation of a non-prescription change in diet or prescription of a GI-specific diet as documented in the EPR), antiparasitic and/or gastrointestinal agent (including antacids, gastro-protectants and antiemetics).	“Prescribed” or “Not prescribed”.
Clinical resolution	No revisit for the diarrhoea episode (or documented resolution at subsequent visit) within 30 days of first presentation. Revisit defined as a revisit for the diarrhoea episode with evidence diarrhoea not resolved.	“Clinical resolution” or “No clinical resolution”.
Treatment escalation	Time (days) from first presentation of acute diarrhoea (prescribed treatment or not) to next presentation when treatment prescribed (further prescription of the same treatment or a different treatment).	Time included as a continuous variable.

### Target trial specification and emulation

Separate target trials were specified and emulated using EPR data to answer the two research questions of interest: 1. “Does antimicrobial prescription relative to no antimicrobial prescription at first presentation with acute diarrhoea in dogs cause a difference in clinical resolution and time to treatment escalation?” and 2. “Does gastrointestinal nutraceutical prescription relative to no gastrointestinal nutraceutical prescription at first presentation with acute diarrhoea in dogs cause a difference in clinical resolution and time to treatment escalation?” [4, 49, 50]. The protocols of the target trials, and the trial emulation, are summarised in Table 2.

**Table 2:** Specification and emulation of target trials to estimate the effect of a) antimicrobial versus no antimicrobial and b) gastrointestinal nutraceutical versus no gastrointestinal nutraceutical prescription for acute diarrhoea in dogs on clinical resolution and time to treatment escalation as the outcomes.

Protocol Component	Target trial description	Emulated trial using veterinary electronic patient records
Research questions	To estimate the effect of: 1. Antimicrobial prescription at first presentation of acute diarrhoea (versus no prescription) on clinical resolution and time to treatment escalation. 2. Gastrointestinal nutraceutical prescription at first presentation of acute diarrhoea (versus no prescription) on clinical resolution and time to treatment escalation.	To estimate the effect of: Antimicrobial prescription and separately gastrointestinal nutraceutical prescription on (i) no revisit within 30 days of first presentation for acute diarrhoea (or evidence of clinical resolution at the subsequent visit) used as a proxy for clinical resolution and (ii) time to treatment escalation.
Eligibility criteria	Dogs aged ≥ 3 months and < 10 years first diagnosed with acute diarrhoea during 2019. Ages to enrol based on consultation with internal medicine specialists and previous clinical trial reports [20, 21, 23]. Exclusion criteria: • Acute diarrhoea due to acute haemorrhagic diarrhoea syndrome, parvovirus, endocrinopathy (including an acute flare-up of a chronic condition), liver disease, kidney disease, immune-mediated disease, pancreatitis, parasitic cause or non-steroidal anti-inflammatory drug (NSAID) use. • Antimicrobial or gastrointestinal nutraceutical prescription within 30 days prior to date of acute diarrhoea diagnosis. • Diarrhoea for greater than 7 days prior to first diagnosis. • Clinical dehydration (defined by admission for intravenous fluids). • Hospitalisation at first presentation for acute diarrhoea. • Death or euthanasia at first presentation for acute diarrhoea.	Same as target trial.
Treatment strategies	1. Antimicrobial prescription or no antimicrobial prescription at first presentation for diarrhoea. 2. Gastrointestinal nutraceutical prescription or no gastrointestinal nutraceutical prescription at first presentation for diarrhoea.	Receive antimicrobial prescription or no antimicrobial prescription at first presentation for acute diarrhoea (with or without any additional medication or dietary modification). Receive nutraceutical prescription or no nutraceutical prescription at first presentation for acute diarrhoea (with or without any additional medication or dietary modification).
Assignment procedures	Eligible dogs will be randomly assigned to either strategy at diagnosis. Owners and veterinarians involved in the dog’s care will be aware of the strategy to which they have been assigned.	Dogs are non-randomly and retrospectively assigned to a treatment strategy. All observed confounding factors will be adjusted for to ensure exchangeability of the groups defined by initiation of the treatment strategies.
Follow-up period	Follow up starts at enrolment (which happens when dogs first present for acute diarrhoea), equivalent to treatment assignment and ends at 30 days.	Same as target trial.
Censoring	Loss to follow up, death, or administrative censoring.	Same as target trial.
Outcome	Primary outcome–time to clinical resolution. Secondary outcome–time to treatment escalation.	Primary outcome–clinical resolution (no revisit with ongoing diarrhoea within 30 days of first presentation). Secondary outcome–time to treatment escalation.
Causal contrasts of interest	Intention to treat effect.	Same as target trial.
Estimands	Risk differences in clinical resolution at follow-up between arms. Causal probabilities for time to treatment escalation.	Risk differences in clinical resolution at follow-up between arms. Causal probabilities for time to treatment escalation.
Analysis plan	Intention to treat analysis, including dogs in each treatment strategy at baseline.	Clinical resolution evaluated as a binary outcome with adjustment for baseline covariates using inverse probability of treatment weighting. Causal time-to-event analysis for time to treatment escalation with adjustment for baseline covariates using inverse probability of treatment weighting.
Adjustment variables	Age at first diagnosis of acute diarrhoea, breed, bodyweight, insurance status, co-morbidity, concurrent clinical signs, duration, severity and vet group at baseline adjusted for through randomisation.	Age at first diagnosis of acute diarrhoea, breed, bodyweight, insurance status, co-morbidity, concurrent clinical signs (vomiting, reduced appetite and pyrexia), other medication prescribed at first presentation, duration, severity (characterised by haematochezia) and vet group.

### Descriptive analysis

Demographic data were described. Continuous variables were assessed graphically for their distribution and summarised using median, interquartile range (IQR) and range given most of them were non-normally distributed. Chi-square test was used to compare categorical variables and the Student’s t-test or Mann–Whitney U test for univariable comparison of continuous variables between groups as appropriate [51].

#### Statistical analysis of the emulated trials

Clinical resolution and time to treatment escalation were compared in a) dogs prescribed antimicrobials and dogs not prescribed antimicrobials for acute diarrhoea and b) dogs prescribed gastrointestinal nutraceuticals and dogs not prescribed gastrointestinal nutraceuticals for acute diarrhoea. For the analysis to be causal, the four assumptions of consistency, no interference, positivity and no unobserved confounding should hold [50]. The consistency assumption implies that an individual’s potential outcome under their observed exposure is the outcome that will actually be observed for that individual [52] i.e. the values of treatment under comparison correspond to well-defined interventions that correspond to the versions of treatment in the data [50]. No interference refers to the assumption that the potential outcomes of one individual are unaffected by the treatment assignment of other individuals [53]. Positivity refers to the assumption that the probability of receiving each treatment conditional on measured covariates is greater than zero [50]. Positivity violations occur when certain subgroups (defined by a combination of covariates) in a sample rarely or never receive some treatments of interest [54].

To emulate randomisation at baseline (diagnosis with acute diarrhoea), the following variables needed to be balanced between treatment groups: age, breed, bodyweight, insurance status, comorbidities, vomiting, reduced appetite, haematochezia, pyrexia, duration, additional treatment prescription and veterinary group attended (as defined and categorised in Table 1 and based on the DAG in Fig 1). We assume that we have adjusted for enough variables to control for confounding. This is a strong assumption and there may be some confounders in a study that are unknown or not measured and hence unobserved.

To evaluate clinical resolution as an outcome, inverse probability of treatment weighting (IPTW) was used. IPTW is a propensity-score based method, with the propensity-score defined as the probability of treatment assignment conditional on observed baseline characteristics [55]. For IPTW, a pseudo-population is created by weighting each individual in the population by the inverse of the conditional probability of receiving the treatment level they indeed received [50]. The goal is to balance covariates between the two treatment groups [56].

To derive the weights using IPTW, a binary logistic regression model was fitted for each trial, with treatment (antimicrobial versus no antimicrobial prescription and gastrointestinal nutraceutical versus no gastrointestinal nutraceutical prescription) as the respective outcome regressed on the confounding variables described above (Table 1). Biologically plausible interaction terms were added to the model and their effect on the standardised mean differences (defined below) were assessed for inclusion, with interaction terms included if they improved covariate balance [57]. The linearity to the logit assumption was assessed by visually inspecting the scatter plot between the continuous predictor (age) and the logit values [58]. The model generated predicted probabilities for each dog of receiving either treatment, which were then used to calculate stabilised IP weights [59]. Extreme weights result when a treated patient has an extremely low or high propensity score but extreme weights can lead to potentially biased results by increasing the variability of the estimated treatment effect. The use of stabilised weights can be achieved by replacing the numerator (which is 1 in the unstabilised weights) with the crude probability of exposure (i.e. given by the propensity score model without covariates) [60, 61]. These weights were then used to weight each dog’s contribution to binary logistic regression outcome models for the antimicrobial and gastrointestinal nutraceutical target trials (Hernán and Robins, 2020 [50], Jiménez-Moro and Gómez, 2014 [62], Robins and Finkelstein, 2000 [63]), with clinical resolution (i.e. no revisit with ongoing diarrhoea within 30 days) as the outcome. The robust (or sandwich) variance estimator was used to obtain valid standard errors [64].

Standardised mean difference (SMD) examines the balance of covariate distribution between treatment groups [65]. For each covariate, SMD between pre- and post-IPTW were calculated, with SMD < 0.1 indicating good covariate balance between the two treatment arms. If SMD is > 0.1 for a variable, the propensity score model should first be re-assessed for improvement e.g. inclusion of further interaction terms, or the variable adjusted for in both the propensity score model and the outcome model to help further adjust for residual confounding [66, 67]. Effect modification was assessed by adding biologically plausible interaction terms to the outcome models and evaluating their effect on the confidence intervals, Akaike information criterion (AIC) and covariate balance (see S1 and S2 Tables in S1 File for results).

To evaluate time to treatment escalation in a) dogs prescribed antimicrobials versus dogs not prescribed antimicrobials for acute diarrhoea and b) dogs prescribed gastrointestinal nutraceuticals and dogs not prescribed gastrointestinal nutraceuticals for acute diarrhoea, unadjusted nonparametric estimation of time-to-event curves was firstly performed. Subsequently, we estimated time-to-event curves via IP weighted hazards models and calculated time-to-event probabilities (and associated 95% Cis) i.e. probability of no treatment escalation at 5-day time intervals [50].

Missing data were handled using the missing-indicator method, which uses a dummy variable in the statistical model to indicate whether the value for that variable is missing [68, 69]. The missing-indicator method assumes that the confounder variable is only a confounder (simultaneously associated with treatment and outcome) when observed, and not when missing. Additionally, we assumed that there is no interaction between the missing indicator and the fully observed confounder in the true propensity score [70]. Data were checked for internal validity and cleaned in Excel (Microsoft Office Excel 2013, Microsoft Corp.), with analyses conducted using R version 4.0.2 (R Core Team, Vienna, Austria). The “IPW” package was used to generate IP weights (and validated manually) [71] (2020). The “survey” package was used for binary logistic regression outcome modelling [72]. The “survival”, “ggplot2” and “survminer” packages were used for the causal time-to-event analysis and associated plots [73–75].

**Fig 2:**
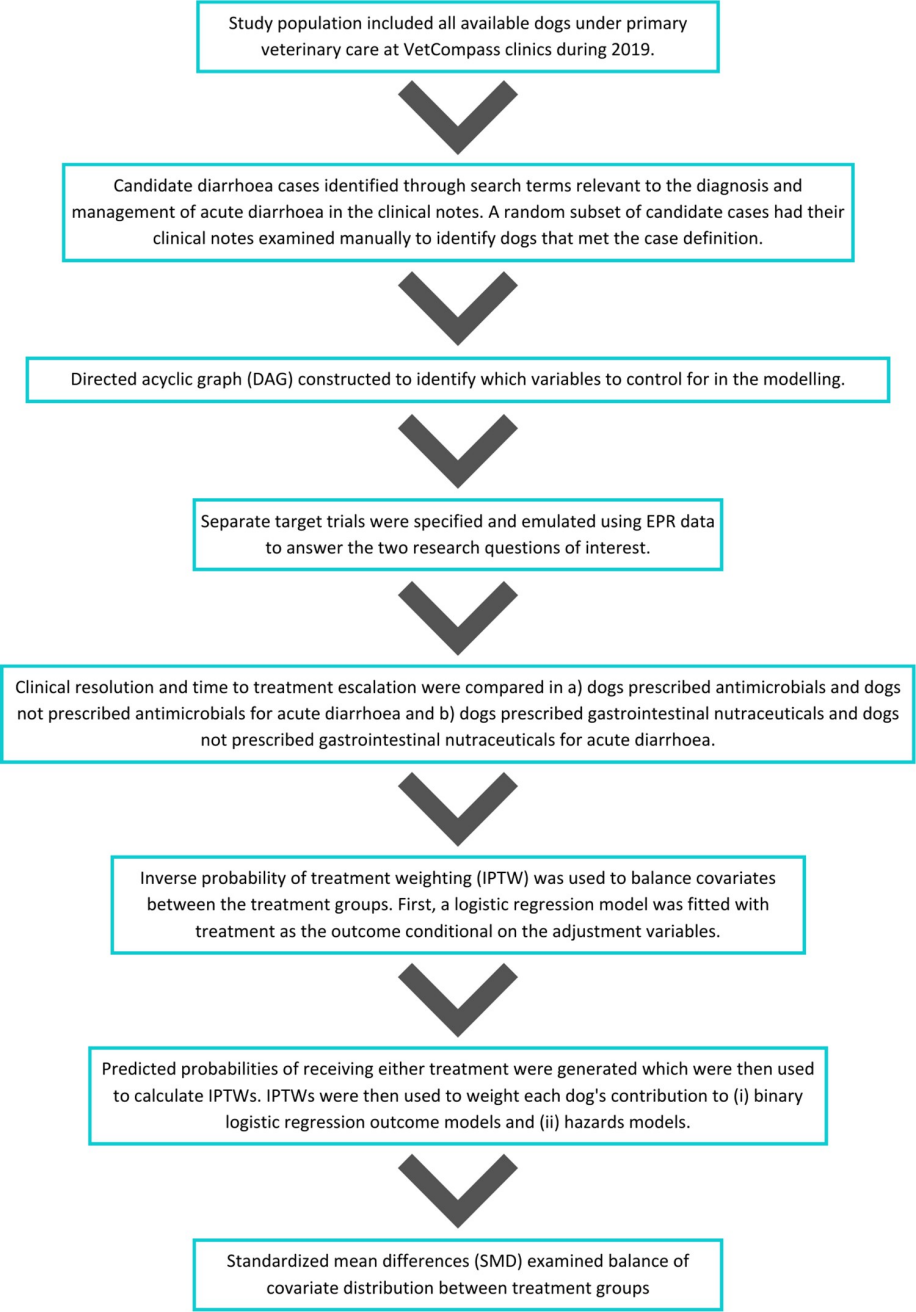
Flowchart depicting the study design and data analysis process.

A flowchart summarising the study design and data analysis process is displayed in Fig 2.

## Results

The study population consisted of 2,250,741 dogs under primary veterinary care in the VetCompass database during 2019. Diarrhoea search terms yielded 808,298 candidate cases, of which 8,080 (1.00%) were manually reviewed. Of these, 894 (11.1%) met the eligibility criteria for the emulated trial. The first emulated trial included: 355 (39.7%) dogs prescribed antimicrobials and 539 (60.3%) dogs not prescribed antimicrobials at first presentation for acute diarrhoea, whilst the second included 597 (66.8%) dogs prescribed gastrointestinal nutraceuticals and 297 (33.2%) dogs not prescribed gastrointestinal nutraceuticals at first presentation for acute diarrhoea. There were 207/894 (23.2%) dogs prescribed both antimicrobials and gastrointestinal nutraceuticals at first presentation for acute diarrhoea.

### Demography of dogs in the emulated trials

Acute diarrhoea cases prescribed antimicrobials had a median age of 3.2 years (IQR 1.4–6.5, range 0.3–9.9) which was older than the median age of cases not prescribed antimicrobials (2.7 years, IQR 1.0–5.7, range 0.3–9.9) (p = 0.005). The median bodyweight of cases prescribed antimicrobials (12.3kg, IQR 7.6–24.6, range 1.1–56.0) did not differ significantly to the median bodyweight of cases not prescribed antimicrobials (11.7kg, IQR 7.3–22.6, range 1.7–70.6) (p = 0.437). The most prevalent breeds among cases prescribed antimicrobials were the Labrador Retriever (7.6% of cases prescribed antimicrobials; 27), German Shepherd Dog (5.9%; 21) and Cockapoo (5.1%; 18) in addition to 77 (21.7%) crossbreds. The most prevalent breeds among cases not prescribed antimicrobials were the Labrador Retriever (7.6%; 41), Cockapoo (5.9%; 32) and Staffordshire Bull Terrier (3.2%; 17) in addition to 130 (24.1%) crossbreds (Table 3).

**Table 3:** Antimicrobial count (% of antimicrobial cases) (n = 355) and no antimicrobial count (% of no antimicrobial cases) (n = 539) for category variables recorded in dogs diagnosed with acute diarrhoea attending primary-care veterinary practices in the VetCompass™ programme in the UK (n = 894).

Variable	Category	Antimicrobial no. (%)	No antimicrobial no. (%)
Breed	Crossbred	77 (21.7)	130 (24.1)
	Purebred—other	162 (45.6)	244 (45.3)
	Labrador Retriever	27 (7.6)	41 (7.6)
	German Shepherd Dog	21 (5.9)	14 (2.6)
	Cockapoo	18 (5.1)	32 (5.9)
	Shih-tzu	13 (3.7)	17 (3.2)
	Staffordshire Bull Terrier	11 (3.1)	17 (3.2)
	French Bulldog	9 (2.5)	17 (3.2)
	Jack Russell Terrier	10 (2.8)	11 (2.0)
	Yorkshire Terrier	7 (2.0)	16 (3.0)
Bodyweight (kg)	< 10	118 (33.2)	184 (34.1)
	10 to < 20	76 (21.4)	111 (20.6)
	20 to < 30	53 (14.9)	80 (14.8)
	≥ 30	39 (11.0)	51 (9.5)
	Not recorded	69 (19.4)	113 (21.0)
Insurance	Non–insured	246 (69.3)	370 (68.6)
	Insured	109 (30.7)	169 (31.4)
Comorbidity at first presentation	Yes	50 (14.1)	109 (20.2)
	No	305 (85.9)	430 (79.8)
Concurrent vomiting	Yes	191 (53.8)	247 (45.8)
	No	164 (46.2)	292 (54.2)
Reduced appetite	Yes	108 (30.4)	133 (24.7)
	No	247 (69.6)	406 (75.3)
Haematochezia	Yes	49 (13.8)	33 (6.1)
	No	306 (86.2)	506 (93.9)
Pyrexia	Yes	52 (14.6)	35 (6.5)
	No	303 (85.4)	504 (93.5)
Duration prior to first presentation	< 24 hours	95 (26.8)	125 (23.2)
	24–48 hours	79 (22.3)	119 (22.1)
	48–96 hours	90 (25.4)	138 (25.6)
	5–7 days	32 (9.0)	58 (10.8)
	Not recorded	59 (16.6)	99 (18.4)
Gastrointestinal nutraceutical prescription	Yes	207 (58.3)	390 (72.4)
	No	148 (41.7)	149 (27.6)
Dietary modification advice	Yes	132 (37.2)	311 (57.7)
	No	223 (62.8)	228 (42.3)
Antiparasitic	Yes	41 (11.5)	70 (13.0)
	No	314 (88.5)	469 (87.0)
Gastrointestinal agent	Yes	156 (43.9)	171 (31.7)
	No	199 (56.1)	368 (68.3)
Veterinary Group	1	126 (35.5)	162 (30.1)
	2	88 (24.8)	183 (34.0)
	3	4 (1.1)	17 (3.2)
	4	70 (19.7)	81 (15.0)
	5	67 (18.9)	96 (17.8)

Acute diarrhoea cases prescribed gastrointestinal nutraceuticals had a median age of 2.7 years (IQR 1.0–6.0, range 0.3–9.9), which did not differ significantly to the median age of cases not prescribed gastrointestinal nutraceuticals (3.2 years, IQR 1.2–6.2, range 0.3–9.9) (p = 0.172). The median bodyweight of cases prescribed gastrointestinal nutraceuticals (11.8kg, IQR 7.3–24.4, range 1.1–70.6) did not differ significantly to the median bodyweight of cases not prescribed gastrointestinal nutraceuticals (12.6kg, IQR 8.0–22.7, range 2.0–52.0) (p = 0.512). The most prevalent breeds among cases prescribed gastrointestinal nutraceuticals were the Labrador Retriever (7.7% of cases prescribed gastrointestinal nutraceuticals; n = 46), Cockapoo (6.5%; 39) and German Shepherd Dog (3.9%; 23) in addition to 138 (23.1%) crossbreds. The most prevalent breeds among cases not prescribed gastrointestinal nutraceuticals were the Labrador Retriever (7.4%; 22), Shih-tzu (4.4%; 13) and German Shepherd Dog (4.0%; 12) in addition to 69 (23.2%) crossbreds (Table 4).

**Table 4:** Gastrointestinal nutraceutical count (% of gastrointestinal nutraceutical cases) (n = 597) and no gastrointestinal nutraceutical count (% of no gastrointestinal nutraceutical cases) (n = 297) for category variables recorded in dogs diagnosed with acute diarrhoea attending primary-care veterinary practices in the VetCompass™ programme in the UK (n = 894).

Variable	Category	Gastrointestinal nutraceutical no. (%)	No gastrointestinal nutraceutical no. (%)
Breed	Crossbred	138 (23.1)	69 (23.2)
	Purebred—other	266 (44.6)	140 (47.1)
	Labrador Retriever	46 (7.7)	22 (7.4)
	German Shepherd Dog	23 (3.9)	12 (4.0)
	Cockapoo	39 (6.5)	11 (3.7)
	Shih-tzu	17 (2.8)	13 (4.4)
	Staffordshire Bull Terrier	21 (3.5)	7 (2.4)
	French Bulldog	17 (2.8)	9 (3.0)
	Jack Russell Terrier	10 (1.7)	11 (3.7)
	Yorkshire Terrier	20 (3.4)	3 (1.0)
Bodyweight (kg)	< 10	205 (34.3)	97 (32.7)
	10 to < 20	128 (21.4)	59 (19.9)
	20 to < 30	88 (14.7)	45 (15.2)
	≥ 30	60 (10.1)	30 (10.1)
	Not recorded	116 (19.4)	66 (22.2)
Insurance	Non–insured	408 (68.3)	208 (70.0)
	Insured	189 (31.7)	89 (30.0)
Comorbidity at first presentation	Yes	103 (17.3)	56 (18.9)
	No	494 (82.7)	241 (81.1)
Concurrent vomiting	Yes	278 (46.6)	160 (53.9)
	No	319 (53.4)	137 (46.1)
Reduced appetite	Yes	153 (25.6)	88 (29.6)
	No	444 (74.4)	209 (70.4)
Haematochezia	Yes	60 (10.1)	22 (7.4)
	No	537 (89.9)	275 (92.6)
Pyrexia	Yes	52 (8.7)	35 (11.8)
	No	545 (91.3)	262 (88.2)
Duration prior to first presentation	< 24 hours	138 (23.1)	82 (27.6)
	24–48 hours	142 (23.8)	56 (18.9)
	48–96 hours	173 (29.0)	55 (18.5)
	5–7 days	50 (8.4)	40 (13.5)
	Not recorded	94 (15.7)	64 (21.5)
Antimicrobial prescription	Yes	207 (34.7)	148 (49.8)
	No	390 (65.3)	149 (50.2)
Dietary modification advice	Yes	321 (53.8)	122 (41.1)
	No	276 (46.2)	175 (58.9)
Antiparasitic	Yes	86 (14.4)	25 (8.4)
	No	511 (85.6)	272 (91.6)
Gastrointestinal agent	Yes	204 (34.2)	123 (41.4)
	No	393 (65.8)	174 (58.6)
Veterinary Group	1	200 (33.5)	88 (29.6)
	2	183 (30.7)	88 (29.6)
	3	14 (2.3)	7 (2.4)
	4	94 (15.7)	57 (19.2)
	5	106 (17.8)	57 (19.2)

### Descriptive analysis of outcomes

The following descriptive analysis of clinical outcomes reports confounded estimates i.e., prior to adjusting for confounding. A similar proportion of dogs prescribed antimicrobials clinically resolved compared with dogs not prescribed antimicrobials (88.5% versus 87.4%, risk difference (RD) 1.1%). Likewise, a similar proportion of dogs prescribed gastrointestinal nutraceuticals clinically resolved compared with dogs not prescribed gastrointestinal nutraceuticals (88.3% versus 86.9%, RD 1.4%) (Table 5).

**Table 5:** Count (%) of treatment prescribed, count (%) and unadjusted risk difference (RD) of clinical resolution of acute diarrhoea in dogs attending primary-care veterinary practices in the VetCompass programme in the UK (n = 894). The RD estimates represent the risk in dogs treated compared with dogs not treated.

Treatment	Prescription at first presentation	Count (%)	Clinical resolution (%)	Unadjusted RD (%)
Antimicrobials	Yes	355 (39.7)	314 (88.5)	1.1
	No	539 (60.3)	471 (87.4)	
Gastrointestinal nutraceuticals	Yes	597 (66.8)	527 (88.3)	1.4
	No	297 (33.2)	258 (86.9)	

A similar proportion of dogs prescribed antimicrobials at first presentation for acute diarrhoea (38; 10.7%) had a treatment escalation compared with dogs not prescribed antimicrobials at first presentation (62; 11.5%). Likewise, a similar proportion of dogs prescribed gastrointestinal nutraceuticals had a treatment escalation (68; 11.4%) compared with dogs not prescribed gastrointestinal nutraceuticals (32; 10.8%). There were 9/894 (1.0%) dogs in both treatment trials with evidence of non-resolution, but these dogs were not prescribed further treatment. The median time (days) to treatment escalation in dogs prescribed antimicrobials (2.0, IQR 1.0–3.8, range 0.0–22.0) did not differ significantly to the median time to treatment escalation in dogs not prescribed antimicrobials (2.5, IQR 1.0–4.8, range 0.0–22.0) (p = 0.280). Conversely, the median time (days) to treatment escalation in dogs prescribed gastrointestinal nutraceuticals (3.0, IQR 1.0–5.0, range 0.0–22.0) was significantly longer than the time to treatment escalation in dogs not prescribed gastrointestinal nutraceuticals (1.0, IQR 0.8–2.3, range 0.0–22.0) (p = 0.004). There were 3/894 (0.3%) dogs that had a revisit for diarrhoea within 12 hours of their initial appointment.

**Fig 3:**
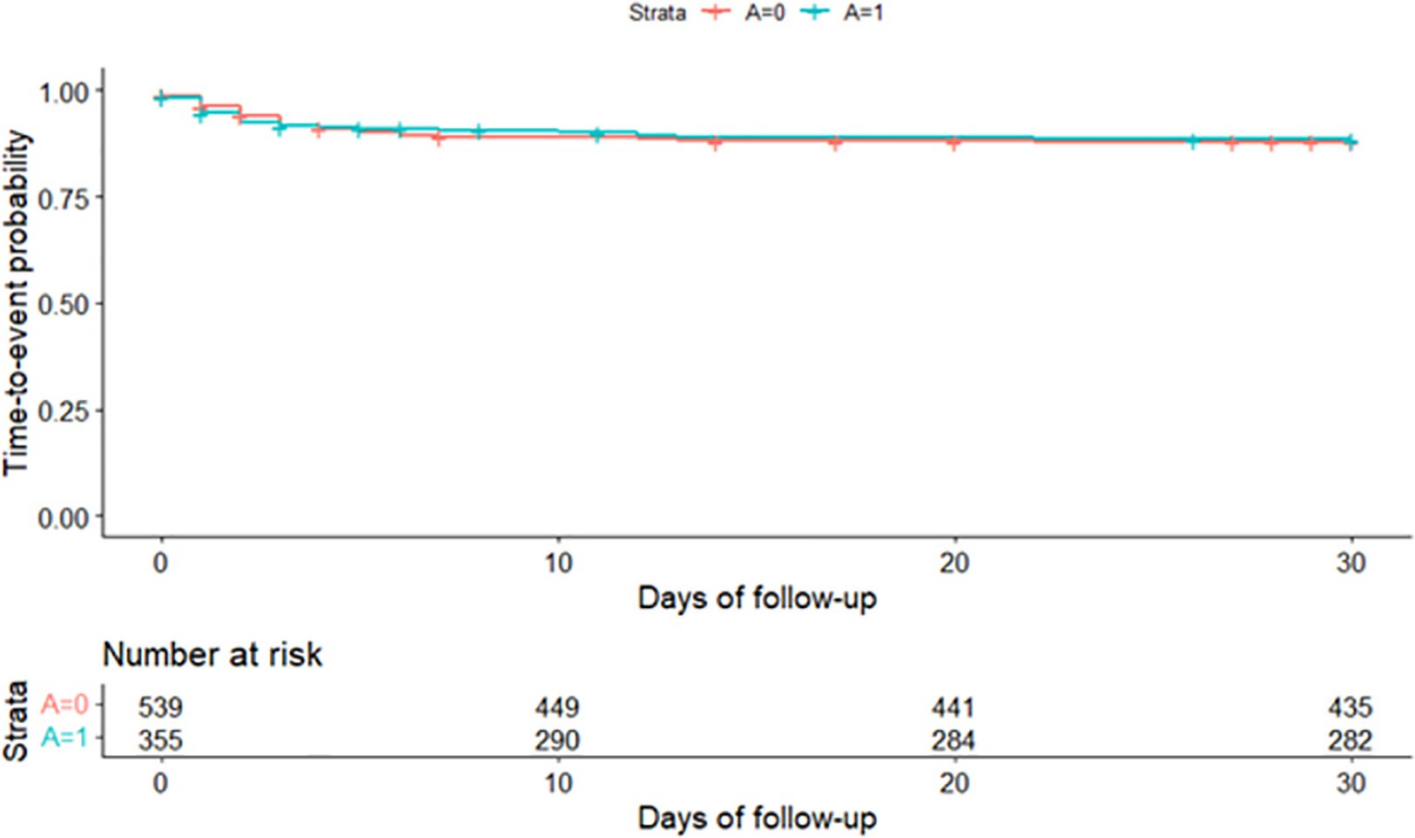
Nonparametric estimation of time-to-event curves for treatment escalation in dogs attending primary-care practices in the UK prescribed antimicrobials (A = 1) and dogs not prescribed antimicrobials (A = 0) at first presentation for acute diarrhoea.

Comparing unadjusted, and therefore potentially confounded, time-to-event from date of first presentation for acute diarrhoea to date of treatment escalation identified no significant difference in time-to-event in antimicrobial cases compared with no antimicrobial cases (log-rank test, P = 0.821) (Fig 3).

**Fig 4:**
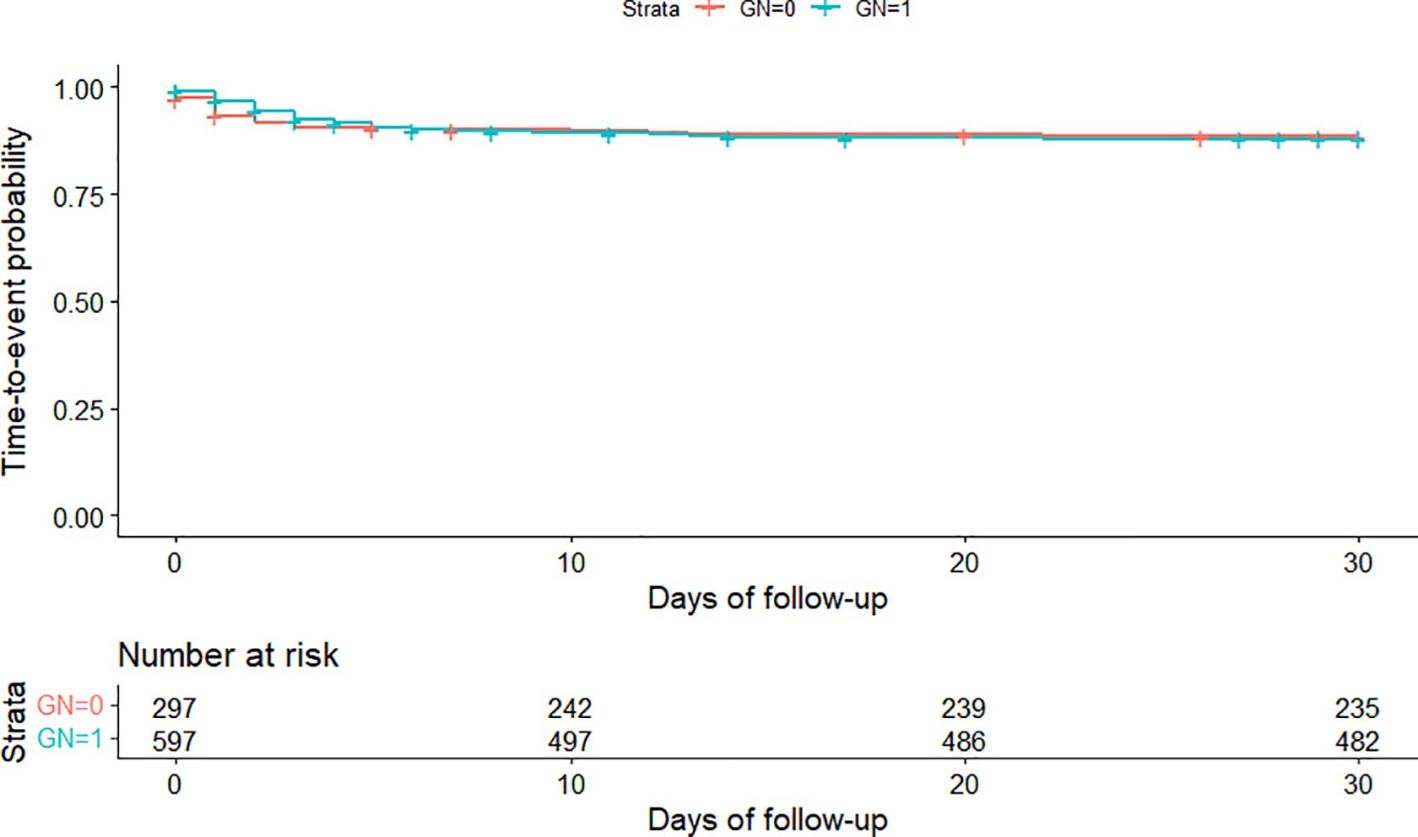
Nonparametric estimation of time-to-event curves for treatment escalation in dogs attending primary-care practices in the UK prescribed gastrointestinal nutraceuticals (GN = 1) and dogs not prescribed gastrointestinal nutraceuticals (GN = 0) at first presentation for acute diarrhoea.

Comparing unadjusted time-to-event from date of first presentation for acute diarrhoea to date of treatment escalation identified no significant difference in time-to-event in gastrointestinal nutraceutical cases compared with no gastrointestinal nutraceutical cases (log-rank test, P = 0.493) (Fig 4).

### Emulated trial results

The final model in the antimicrobial prescription trial included the following covariates to generate propensity scores: age (including a quadratic term), breed, bodyweight, insurance status, comorbidities, vomiting, age*vomiting (* indicating interaction), reduced appetite, haematochezia, pyrexia, haematochezia*pyrexia, duration, veterinary group, gastrointestinal nutraceutical prescription, dietary modification advice, gastrointestinal nutraceutical prescription*dietary modification advice, antiparasitic and gastrointestinal agent. The final model in the gastrointestinal nutraceutical prescription trial included the following covariates to generate propensity scores: age (including a quadratic term), breed, bodyweight, insurance status, comorbidities, vomiting, reduced appetite, haematochezia, age*haematochezia, pyrexia, duration, veterinary group, antimicrobial prescription, dietary modification advice, antiparasitic and gastrointestinal agent.

After balancing covariates between the antimicrobial and no antimicrobial prescribed dogs using IPTW, antimicrobial prescription caused no significant difference in clinical resolution compared with no antimicrobial prescription. Specifically, the risk difference for clinical resolution in dogs prescribed antimicrobials versus no antimicrobials was 0.4% (95% CI -4.5 to 5.3). Likewise, after balancing covariates between the gastrointestinal nutraceutical and non-gastrointestinal nutraceutical prescribed dogs using IPTW, gastrointestinal nutraceutical prescription caused no significant difference in clinical resolution compared with non-gastrointestinal nutraceutical prescription. Specifically, the risk difference for clinical resolution in dogs prescribed gastrointestinal nutraceuticals versus non-gastrointestinal nutraceuticals was 0.3% (95% CI -4.5 to 5.0). (Table 6).

**Table 6:** Risk difference (RD) (and 95% confidence intervals [CI]) for clinical resolution in dogs under UK primary veterinary care prescribed antimicrobials or gastrointestinal nutraceuticals for acute diarrhoea. The estimates represent the risk in dogs prescribed antimicrobials or gastrointestinal nutraceuticals using inverse probability of treatment weighting to adjust for confounding.

Treatment	Adjusted risk of clinical resolution as if all dogs prescribed (%)	Adjusted risk of clinical resolution as if all dogs not prescribed (%)	IPW RD
Antimicrobials	88.3	87.9	0.4% (95% CI -4.5% to 5.3%)
Gastrointestinal nutraceuticals	88.2	88.0	0.3% (95% CI -4.5% to 5.0%)

### Time-to-event analysis for treatment escalation

#### Antimicrobial trial

**Fig 5:**
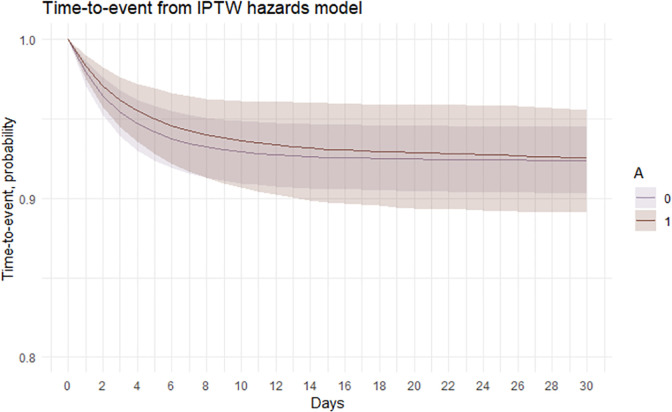
Estimation of time-to-event curves via IP weighted hazards model (and 95% confidence intervals calculated via bootstrapping) for treatment escalation in dogs attending primary-care practices in the UK prescribed antimicrobials (A = 1) and dogs not prescribed antimicrobials (A = 0) at first presentation for acute diarrhoea.

Following adjustment for confounding using an IP weighted hazards model, time-to-event curves (and corresponding 95% CIs) were estimated (Fig 5) i.e., the probability of not having a treatment escalation over time.

Time-to-event probabilities, i.e., the probability of no treatment escalation, were calculated from day 1 to 30 at regular time intervals (Table 7). The probabilities as if all dogs were treated with antimicrobials at first presentation for acute diarrhoea and the probabilities as if all dogs were not treated with antimicrobials were computed. The time-to-event probabilities marginally decreased over time in both groups, with a narrow difference in time-to-event probability (range 0.11–0.74%), with overlap in 95% confidence intervals at all time points.

**Table 7:** Time-to-event probabilities and difference in probability (with 95% confidence intervals) in dogs attending primary-care practices in the UK as if all dogs prescribed antimicrobials and as if all dogs not prescribed antimicrobials at first presentation for acute diarrhoea, with treatment escalation as the outcome. Time-to-event probabilities were estimated via IP weighted hazards model.

Days after first presentation for acute diarrhoea	Time-to-event probability as if all dogs prescribed antimicrobials (%)	Time-to-event probability as if all dogs not prescribed antimicrobials (%)	Difference in time-to-event probability (%)	95% Confidence Intervals
1	98.29	97.88	0.41	-0.70 to 1.59
5	94.86	94.12	0.74	-1.92 to 3.46
10	93.49	92.93	0.56	-2.81 to 3.91
15	93.00	92.60	0.40	-3.28 to 3.97
20	92.75	92.47	0.28	-3.53 to 4.00
25	92.60	92.40	0.20	-3.67 to 4.01
30	92.46	92.35	0.11	-3.86 to 3.89

#### Gastrointestinal nutraceutical trial

**Fig 6:**
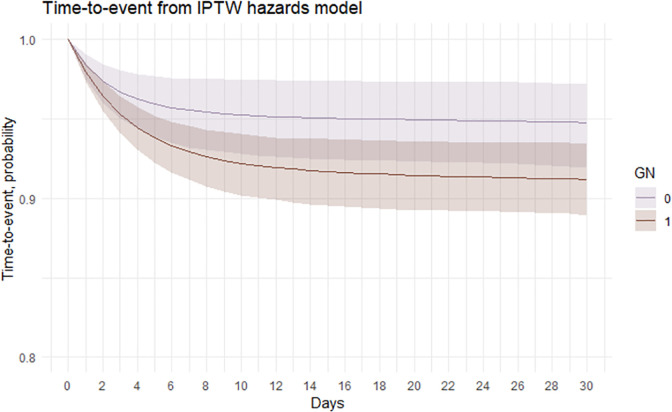
Estimation of time-to-event curves via IP weighted hazards model (and 95% confidence intervals calculated via bootstrapping) for treatment escalation in dogs attending primary-care practices in the UK prescribed gastrointestinal nutraceuticals (G = 1) and dogs not prescribed gastrointestinal nutraceuticals (G = 0) at first presentation for acute diarrhoea.

Following adjustment for covariates using an IP weighted hazards model, time-to-event curves (and corresponding 95% CIs) were estimated (Fig 6) i.e., the probability of not having a treatment escalation over time.

Time-to-event probabilities and difference in probability were calculated from day 1 to 30 at regular time intervals (Table 8). The probabilities as if all dogs were treated with gastrointestinal nutraceuticals at first presentation for acute diarrhoea and the probabilities as if all dogs were not treated with gastrointestinal nutraceuticals were computed. The time-to-event probabilities marginally decreased over time in both groups, with a narrow difference in time-to-event probability, ranging from -0.42 to -3.50%. The confidence intervals were all below 0 from day 10 onwards, indicating that prescribing gastrointestinal nutraceuticals to all dogs at the first presentation for acute diarrhoea, it would cause an increase in treatment escalation from day 10 onwards compared with dogs not prescribed gastrointestinal nutraceuticals at first presentation.

**Table 8:** Time-to-event probabilities and difference in probability (with 95% confidence intervals) in dogs attending primary-care practices in the UK as if all dogs prescribed gastrointestinal nutraceuticals and as if all dogs not prescribed gastrointestinal nutraceuticals at first presentation for acute diarrhoea, with treatment escalation as the outcome. Time-to-event probabilities were estimated via IP weighted hazards model.

Days after first presentation for acute diarrhoea	Time-to-event probability as if all dogs prescribed gastrointestinal nutraceuticals (%)	Time-to-event probability as if all dogs not prescribed gastrointestinal nutraceuticals (%)	Difference in time-to-event probability (%)	95% confidence intervals
1	97.94	98.36	-0.42	-1.34 to 0.56
5	93.81	95.85	-2.04	-4.56 to 0.16
10	92.20	95.20	-3.00	-5.97 to -0.23
15	91.65	95.01	-3.36	-6.49 to -0.41
20	91.42	94.92	-3.50	-6.79 to -0.40
25	91.29	94.83	-3.54	-6.92 to -0.41
30	91.20	94.69	-3.49	-6.97 to -0.35

## Discussion

Interest in the target trial emulation framework is gaining momentum in human epidemiology [76], with the current study being one of the pioneering applications of target trial emulation in the veterinary field. The framework aims to make the ‘target trial’ explicit, which helps avoid common design flaws and methodological pitfalls in the analysis of non-randomised studies, keeping each step transparent and accessible [4]. Indeed, studies in the human medical literature have demonstrated that it is possible to approximate the results from well conducted RCTs using the target trial framework, combined with using suitable analytical methods [77–82]. Therefore, this is a powerful approach, which the current study has demonstrated can be successfully applied to veterinary EHR data.

After balancing covariates between the antimicrobial and no antimicrobial groups using IPTW, antimicrobial prescription at first presentation of acute diarrhoea in dogs did not cause a significant change (ie neither an increase or decrease) in proportional clinical resolution (no revisit with ongoing diarrhoea within 30 days of first presentation) (RD 0.4%, 95% CI -4.5% to 5.3%) compared with no antimicrobial prescription. Time-to-event analysis similarly identified no significant difference in the probability of treatment escalation between dogs prescribed antimicrobials and dogs not prescribed antimicrobials at first presentation of acute diarrhoea. Therefore, these results provide no evidence supporting a difference in “clinical resolution” risk or time to treatment escalation in dogs prescribed antimicrobials versus not prescribed antimicrobials at first presentation of acute diarrhoea.

These findings are in line with those of a large observational study [18] and previous clinical trial reports, which also did not identify a significant difference in clinical resolution between dogs prescribed antimicrobials and dogs not prescribed antimicrobials [20–23]. The current results provide further evidence to support the British Small Animal Veterinary Association (BSAVA) “PROTECT ME” guidelines, which state that dogs presenting with acute gastrointestinal signs, including dogs with haemorrhagic diarrhoea that are systemically well, do not require antimicrobial therapy [83]. Likewise, the results support European antimicrobial stewardship guidelines (ASGs), as reported in a recent review, where all evaluated ASGs did not recommend antimicrobials for treatment of acute diarrhoea in dogs [14]. Given a relatively high proportion of dogs were prescribed antimicrobials at first presentation of acute diarrhoea in the current study (39.7%), there still appears to be a disconnect between guidance and clinical practice. That said, the proportion treated in this 2019 cohort is lower than a previous report of 52.5% dogs with acute uncomplicated diarrhoea prescribed antimicrobials within 10 days of first veterinary presentation from 2014–2017 [18]. Therefore, this could represent moves within the veterinary profession towards decreasing prescription of antimicrobials for acute diarrhoea over time, although different study designs and case definitions may in part explain this variation.

After balancing covariates between the gastrointestinal nutraceutical and no gastrointestinal nutraceutical treatment groups using IPTW, gastrointestinal nutraceutical prescription at first presentation of acute diarrhoea in dogs did not cause a significant increase or decrease in clinical resolution compared with no gastrointestinal nutraceutical prescription. Time-to-event analysis identified that if all dogs were prescribed gastrointestinal nutraceuticals at first presentation for acute diarrhoea, it would cause an increase in treatment escalation from day 10 onwards compared with dogs not prescribed gastrointestinal nutraceuticals at first presentation. However, although significant, the difference in time-to-event probability was minor.

The current study findings of no significant difference in “clinical resolution” of acute diarrhoea between dogs prescribed gastrointestinal nutraceuticals and dogs not prescribed gastrointestinal nutraceuticals fits with a previous systematic review. That systematic review pointed toward a very limited and possibly clinically unimportant effect for gastrointestinal nutraceuticals in the prevention or treatment of acute gastrointestinal disease [37]. A previous large-scale observational study based on primary-care data found that prescription of gastrointestinal nutraceuticals, in combination with dietary modification, were positively associated with resolution of signs [18]. It is possible that dietary modification is the overriding factor for clinical resolution, rather than gastrointestinal nutraceuticals. The current study collected data on “dietary modification advice” as an adjustment variable, however the specific diet fed will vary and it is unknown to what extent the advice was heeded. Further prospective studies evaluating diet as a primary exposure might help to clarify the contribution of dietary modification in clinical resolution of acute diarrhoea.

Although minimal, dogs prescribed gastrointestinal nutraceuticals at first presentation of acute diarrhoea were more likely to have a treatment escalation than dogs not prescribed gastrointestinal nutraceuticals from day 10 onwards. Gastrointestinal nutraceuticals are often prescribed as a first-line treatment for acute diarrhoea [18], therefore, this might indicate that owners of dogs prescribed gastrointestinal nutraceuticals at first presentation exhibit health-seeking behaviour and are more invested in a clinical response. Further studies, including qualitative studies exploring owner expectations for acute diarrhoea treatment and resolution, might help to clarify the effect identified in the current study.

The limitations of this study are largely based on the nature of retrospective analysis of electronic patient record data, including issues related to unobserved confounding, missing and misclassified data and application of a case definition to the data available [84]. Although analysis of VetCompass EPR data can generate evidence generalisable to the overall dog population, case definitions, diagnosis recording and history-taking relies on the clinical acumen and note-taking of attending practitioners, with no attempts made by the researchers to second-guess diagnoses [84]. “Clinical resolution” is difficult to define within retrospective primary-care veterinary EPR data, especially for an acute and often self-limiting condition. Clinical resolution defined as no revisit with ongoing diarrhoea within 30 days of first presentation was reasoned as an appropriate outcome, in line with a previous study based on primary-care EPR data [18]. Dogs might have been prescribed treatments other than antimicrobials and gastrointestinal nutraceuticals which could affect outcome, however concurrent treatments were included as covariates to help account for these differences. Expert opinion was sought in construction of the DAG, however it is possible unmeasured confounders could influence the risk differences and time-to-event probabilities calculated.

Causal inference methods, and specifically the target trial emulation framework presented in this study, focus on the relationship between exposure and outcome [85]. We described the variables collected as covariates (as identified in the DAG) for clarity, however we did not specifically analyse confounder effects to avoid “Table 2 Fallacy” [86]. This being that presenting multiple estimated effect measures from the same model encourages the reader to interpret all these estimates in the same way, however the interpretation of a confounder effect estimate may be different than for the exposure effect estimate, due to underlying differences in the causal model [86].

## Conclusions

Overall, this study demonstrated successful application of the target trial framework to veterinary observational data. Acute diarrhoea was used as the condition of interest, with the findings showing that antimicrobial or gastrointestinal prescription at first presentation of acute diarrhoea in dogs causes no difference in proportional clinical resolution (i.e., no revisit with ongoing diarrhoea within 30 days of first presentation). Time-to-event analysis identified no significant difference in the probability of treatment escalation between dogs prescribed antimicrobials and dogs not prescribed antimicrobials at first presentation of acute diarrhoea. Dogs prescribed gastrointestinal nutraceuticals at first presentation of acute diarrhoea were more likely to have a treatment escalation than dogs not prescribed gastrointestinal nutraceuticals from day 10 onwards, however the difference was minimal. Overall, the majority of dogs did not have a revisit for the diarrhoea episode, or a treatment escalation, whether they were prescribed antimicrobials or gastrointestinal nutraceuticals at first presentation or not. These findings support the recommendation to restrict the use of antimicrobials for acute diarrhoea in dogs within the veterinary profession.

## Supporting information

S1 File Model evaluation. (DOCX)

## Abbreviations

AIC: Akaike information criterion
AMR: antimicrobial resistance
ASGs: antimicrobial stewardship guidelines
BSAVA: British Small Animal Veterinary Association
CI: confidence interval
DAG: directed acyclic graph
EPR: electronic patient record
IP: inverse probability
IPTW: inverse probability of treatment weighting
IQR: interquartile range
RCT: randomised controlled trial
RD: risk difference
SMD: standardised mean difference
