# Supplementary material for: Target trial emulation: Do antimicrobials or gastrointestinal nutraceuticals prescribed at first presentation for acute diarrhoea cause a better clinical outcome in dogs under primary veterinary care in the UK?
Source: PLoS One. 2023 Oct 4;18(10):e0291057. doi: 10.1371/journal.pone.0291057 (PMC10550114; doi:10.1371/journal.pone.0291057)
Supplement: S1 File — (DOCX) [file pone.0291057.s001.docx]

Supporting Information

*Model evaluation*

The standardised mean differences between groups for each of the covariates pre and post IPTW are shown in Table 1 and Table 2. The standardised mean differences in the weighted sample were all below 0.1 for each covariate, indicating well-balanced groups post weighting. The median stabilised IP weight for the antimicrobial treatment trial was 0.87 (range 0.41 – 4.71), whilst the median stabilised IP weight for the gastrointestinal nutraceutical treatment trial was 0.92 (range 0.45 to 3.95). The linear assumption for age as a continuous covariate was not met in either trial, therefore a quadratic term was added to account for this. For the antimicrobial treatment trial, interaction terms between haematochezia and pyrexia, age and vomiting and gastrointestinal nutraceutical prescription and dietary modification advice improved the balance. For the gastrointestinal nutraceutical treatment trial, interaction terms between age and haematochezia improved the balance. Significant effect modification was not evident in either trial.

**Table 1: Standardised mean differences (SMD) before and after applying inverse probability weighting for the antimicrobial versus no antimicrobial cases. This table shows the SMD for each of the prespecified covariates pre and post weighting.**

| Variable | SMD before weighting | SMD after weighting |
| --- | --- | --- |
| Bodyweight (kg) | 0.063 | 0.023 |
| Age (years) | 0.161 | 0.029 |
| Insurance status | 0.014 | 0.001 |
| Comorbidity | 0.163 | 0.013 |
| Vomiting | 0.160 | 0.027 |
| Reduced appetite | 0.129 | 0.034 |
| Haematochezia | 0.259 | 0.004 |
| Pyrexia | 0.268 | 0.006 |
| Duration | 0.100 | 0.053 |
| Breed | 0.197 | 0.069 |
| Veterinary group | 0.267 | 0.027 |
| Gastrointestinal nutraceutical prescription | 0.298 | 0.021 |
| Dietary modification advice | 0.420 | 0.005 |
| Antiparasitic | 0.044 | 0.020 |
| Gastrointestinal agent | 0.254 | 0.002 |

**Table 2: Standardised mean differences (SMD) before and after applying inverse probability weighting for the gastrointestinal nutraceutical versus no gastrointestinal nutraceutical cases. This table shows the SMD for each of the prespecified covariates pre and post weighting.**

| Variable | SMD before weighting | SMD after weighting |
| --- | --- | --- |
| Bodyweight (kg) | 0.077 | 0.051 |
| Age (years) | 0.089 | 0.005 |
| Insurance status | 0.037 | <0.001 |
| Comorbidity | 0.042 | 0.014 |
| Vomiting | 0.147 | 0.007 |
| Reduced appetite | 0.090 | 0.020 |
| Haematochezia | 0.094 | 0.028 |
| Pyrexia | 0.102 | 0.004 |
| Duration | 0.332 | 0.051 |
| Breed | 0.265 | 0.059 |
| Veterinary group | 0.114 | 0.073 |
| Antimicrobial prescription | 0.311 | 0.019 |
| Dietary modification advice | 0.189 | 0.009 |
| Antiparasitic | 0.256 | 0.004 |
| Gastrointestinal agent | 0.150 | 0.018 |
